# Appropriate use criteria and the impact of stress cardiovascular magnetic resonance (CMR) imaging on management of patients with known or suspected coronary artery disease

**DOI:** 10.1186/1532-429X-17-S1-O36

**Published:** 2015-02-03

**Authors:** Sloane A McGraw, Omer Mirza, Michael A Bauml, Vibhav Rangarajan, Afshin Farzaneh-Far

**Affiliations:** 1 Cardiology, University of Illinois-Chicago, Chicago, IL, USA; 2 Cardiology, Duke University, Durham, NC, USA

## Background

Stress-CMR provides important diagnostic and prognostic information in patients with known or suspected coronary artery disease. However, in the current fiscal environment, use of a newer imaging modality like stress-CMR requires evidence for direct additive impact on clinical management. Appropriate use criteria (AUC) have recently been developed to provide guidance to physicians and payers regarding the appropriateness of this test in various clinical scenarios. However, these criteria were created by expert consensus and have never been systematically validated. The aims of this study were 1) to evaluate the impact of stress-CMR on clinical management; and 2) to determine the relationship of the AUC with active clinical impact.

## Methods

We prospectively enrolled 247 consecutive outpatients undergoing stress-CMR at a tertiary academic center in the United States. Definitions for "active clinical impact" were pre-defined and assessed from the medical records and/or patients by two cardiologists. The categories of "active clinical impact" were: referral to invasive coronary angiography, clearance for surgery, referral for additional diagnostic testing, subspecialty referral, medication change, and discharge from clinic. Two independent general cardiologists reviewed all clinical information dated before the CMR-stress test. These reviewers were blinded to the results of the CMR and to the clinical course subsequent to the test. The CMR stress tests were classified as "appropriate', "maybe appropriate" or "rarely appropriate" as defined by the 2013 AUC.

## Results

**Figure 1:**
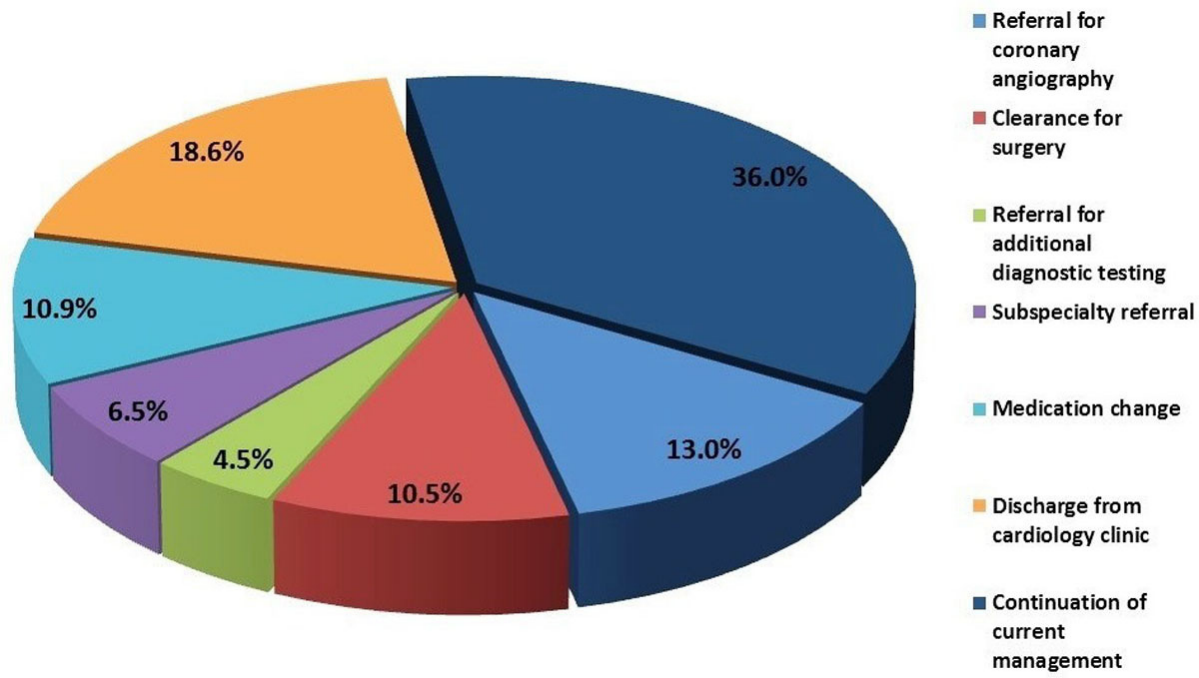
Impact of stress CMR on clinical management.

**Figure 2:**
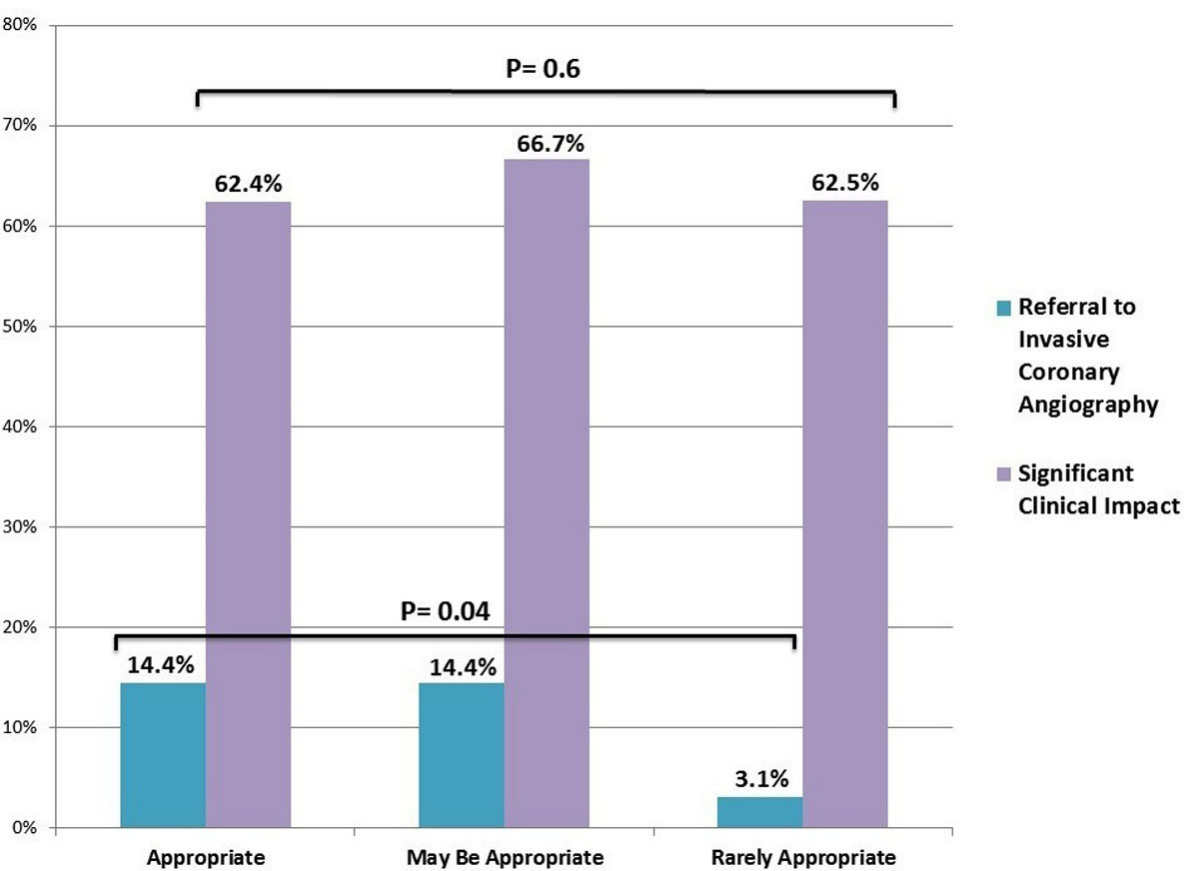
Clinical impact based on the appropriate use criteria.

Overall, stress-CMR resulted in active clinical changes in 64% of patients (Figure 1). This included angiography in 13.0%, clearance for surgery in 10.5%, referral for additional diagnostic testing in 4.5%, subspecialty referral in 6.5%, medication change in 10.9%, and discharge from cardiology clinic in 18.6%. When classified by the AUC, 51% of patients were categorized as "appropriate", 36% as "may be appropriate", and 13% were "rarely appropriate". Overall, the proportion of CMR-stress tests resulting in active clinical change was similar in all 3 AUC categories (62.4%, 66.7%, and 62.5%; p=0.6). However, the proportion of CMR-stress tests resulting in coronary angiography was significantly greater in the "appropriate" (14.4%) and "may be appropriate" (14.4%) groups compared to those classified as "rarely appropriate" (3.1%) (p=0.04) (Figure 2).

## Conclusions

Stress-CMR directly resulted in active clinical change in 64% of patients. Overall, the AUC were a poor predictor of subsequent active clinical change. However, "appropriate" and "may be appropriate" studies more frequently resulted in coronary angiography than those classified as "rarely appropriate."

## Funding

None.

